# Comparing Repetition-Velocity Output from a Motor-Integrated Magnetic Sensing System with That of a Linear Position Transducer Across Resistance Exercises and Loads: A Human Trial Experiment

**DOI:** 10.3390/s26206466

**Published:** 2026-10-13

**Authors:** Alchan Kim

**Affiliations:** Department of Sports & Technology, Seokyeong University, 124 Seogyeong-ro, Seongbuk-gu, Seoul 02713, Republic of Korea; younal@skuniv.ac.kr; Tel.: +82-02-940-7210; Fax: +82-02-940-7616

**Keywords:** equipment-integrated sensing, magnetic position sensing, repetition velocity, resistance exercise, velocity-based training, inter-device agreement, linear position transducer, Bland–Altman analysis, metrological characterization

## Abstract

Equipment with integrated sensing can now provide repetition-velocity feedback without external sensors. However, whether their output agrees with that of established commercial linear position transducers has yet to be investigated. This study addresses this gap by comparing repetition-velocity measurements obtained from the motor-integrated magnetic sensing system of MOTY V with those obtained from GymAware RS to evaluate inter-device agreement and measurement characteristics. Deadlifts and squats were performed at 40%, 60%, and 80% of estimated one-repetition maximum by 15 male students, with both devices simultaneously measuring bar movement. The final analysis included 897 paired repetitions out of 900 planned pairs. Mean velocities were 0.7102 ± 0.1496 m/s for MOTY V and 0.6816 ± 0.1410 m/s for GymAware RS. The mixed-effects mean bias (MOTY V–GymAware RS) was 0.0285 m/s (95% confidence interval [CI], 0.0216–0.0353), with repeated-measures 95% limits of agreement ranging from −0.0773 to +0.1342 m/s. Mean absolute error and root mean square error were 0.0467 and 0.0609 m/s, respectively. Repetition-level correlation was r = 0.933, and set-level absolute-agreement intraclass correlation coefficient (ICC) was 0.958 (95% CI, 0.929–0.973). Mean bias did not differ significantly by exercise, load, or their interaction, whereas proportional bias was significant (β = 0.125, *p* < 0.001). MOTY V showed high agreement with GymAware RS, but small positive systematic and proportional biases preclude assuming direct interchangeability.

## 1. Introduction

In resistance training, external load is an important determinant of mechanical demand and subsequent training adaptations. External load has traditionally been prescribed in training programs as a percentage of the one-repetition maximum (1RM) or the number of repetitions that can be completed with proper form. However, bar-movement velocity at a given relative load may vary with training status, daily readiness, and fatigue. Velocity-based resistance training accounts for this variability by monitoring repetition velocity to inform exercise intensity and load adjustment. For many resistance exercises, movement velocity generally decreases as relative load increases, although the load–velocity relationship is movement-specific and may vary with execution conditions [1,2].

Repetition velocity has also been used to monitor within-set fatigue, execution quality, and training volume. The decline in repetition velocity under a constant load has been associated with neuromuscular fatigue and the level of effort during resistance exercise [2]. Thus, monitoring velocity loss may help inform decisions about set termination or load adjustment. Providing velocity after each repetition may also encourage the individual to maintain maximal concentric velocity [3]. From a practical perspective, this requires a device that measures repetition velocity consistently with an immediate result.

Today, individuals often use linear position transducers, inertial sensors, optical sensors, camera-based systems, and mobile applications to measure their movement velocity during resistance training. Commonly, linear position transducers (LPTs) are used to estimate velocity from the time-varying displacement of a cable attached to the equipment’s bar or weight load. These LPTs have become common tools for measuring resistance-exercise kinematics. One systematic review reported that LPTs provide greater accuracy and reproducibility than other portable monitoring technologies [4]. Previous studies have evaluated the validity and reliability of the GymAware PowerTool (Kinetic Performance Technology Pty Ltd., Canberra, Australia), a cable-based linear position transducer, during common resistance exercises, including squats and deadlifts [5,6]. GymAware RS (Kinetic Performance Technology Pty Ltd., Canberra, Australia) was evaluated separately by Lemus et al. [7], who compared peak concentric velocity against a 12-camera motion-capture system during snatch and clean movements in 12 weightlifters at 40% and 70% of 1RM. Although significant differences were observed across the devices evaluated in that study (*p* < 0.001), GymAware RS showed no statistically significant fixed or proportional bias in peak-velocity measurement. These findings concern peak rather than mean velocity and different exercises from those examined here. GymAware RS was therefore selected as an established commercial comparator rather than an absolute criterion system.

In device-comparison studies, correlation coefficients are frequently reported to quantify the association between measurements. However, a high correlation only indicates that two measurements change in a similar direction. It does not, therefore, reveal whether they yield the same numerical values. Differences in sensing principle, sampling, filtering, repetition segments, and velocity calculations can produce systematic discrepancies, even when the same movement is measured [4]. Thus, although there can be strong correlations between velocity-monitoring devices, they can have systematic differences and wide limits of agreement. Consequently, values or thresholds from one device should not automatically be applied to another [8]. Reproducibility and the sensitivity to detect meaningful changes in performance also vary across commercial velocity-monitoring devices and velocity metrics [9]. Measurement validity can also be influenced by how and where a device is attached to the equipment [10]. Therefore, in addition to correlation, evaluations of new systems should consider mean bias, limits of agreement, absolute agreement, and measurement-error indices.

Motor-driven/digitally controlled resistance-training systems that generate resistance and provide exercise data in real time commonly use sensors to estimate displacement and velocity and control information already entered into the equipment. Previous studies have compared velocity outputs from motorized resistance-training systems with those obtained using external kinematic measurement devices [11,12].

The advantage of equipment with integrated sensing is that these systems can provide repetition-level information without the need for separate external velocity sensors, thereby offering real-time feedback and velocity-based load control. Nevertheless, equipment-integrated sensing alone does not establish measurement validity or inter-device agreement; these characteristics require further independent evaluation. For validity and equipment comparisons, velocity output needs to be evaluated under real-time exercise conditions by simultaneously investigating the external system and quantifying bias, limits of agreement, and measurement error.

MOTY V (MOTY Inc., Seoul, Republic of Korea) is a digitally controlled cable resistance-training machine that measures motor-shaft rotation using an integrated magnetic sensing assembly. It converts rotation to cable displacement and velocity using the effective radius of a directly coupled pulley. The system reports mean concentric velocity for each repetition without an external velocity sensor. Thus, MOTY V derives cable displacement and mean concentric velocity through observer-based signal processing and filtering. In contrast, GymAware RS is a tether-based linear position transducer that derives a manufacturer-defined mean-velocity metric from linear displacement. Because the two systems differ in sensing principle and signal-processing architecture, agreement between their velocity outputs cannot be assumed even when the same movement is measured. However, no study has yet examined whether the output of MOTY V agrees with that of an established commercial LPT, or whether the between-device bias varies by exercise or relative load.

To address this gap, this study evaluated the inter-device agreement and measurement characteristics of repetition-velocity measurements obtained simultaneously from MOTY V and GymAware RS during deadlift and squat exercises performed at different relative loads. The analyses examined mean bias, repeated-measures 95% limits of agreement, absolute-agreement intraclass correlation, mean absolute error, root mean square error, and proportional bias. The hypotheses were that the two machines would have a strong association in repetition velocity and high absolute agreement, and that their mean bias would not differ significantly among exercises or relative loads.

## 2. Materials and Methods

### 2.1. Study Design and Ethical Approval

A repeated-measures sensor study was conducted, comparing the characteristics of the two velocity-measurement systems through a standardized experimental protocol. This study was not designed as an intervention trial to evaluate health or training effects, but was rather designed as a methodological study of inter-device agreement in repetition-velocity measurement.

The study was conducted at the Sporex facility of Seokyeong University in Seoul, Republic of Korea. The protocol was approved by the Institutional Review Board of Seokyeong University (SKUIRB-2025-02-001; 23 May 2025), and all participants provided written informed consent before participation. Data were collected in April 2026.

### 2.2. Participants

Male university students were recruited from Seokyeong University and other universities through convenience sampling and existing research networks. Eligibility criteria included: male sex, university-student status, at least six months of resistance-training experience, and the ability to perform the deadlift and squat exercises. Individuals with any recent musculoskeletal injury, cardiovascular disease, or other condition limiting resistance exercise were excluded. All 15 recruited participants were included in the final analysis, and no participant dropped out. The sample size was determined by the feasible scope of this methodological comparison study, and no a priori sample-size or precision calculation was performed. Each participant was assessed under two exercise conditions and three relative loads, with both devices measuring the same repetitions simultaneously. The resulting 897 paired repetitions represented repeated observations clustered within 15 participants rather than 897 independent experimental units. Participant characteristics are presented in Table 1.

### 2.3. MOTY V Equipment-Integrated Magnetic Sensing System

As noted, MOTY V is a motor-driven cable resistance-training system that derives cable displacement and velocity from a magnetic rotary position-sensing assembly integrated at the end of a motor shaft, without a separate sensor on the cable. The sensing assembly comprises two Infineon TLE4997 linear Hall sensors (Infineon Technologies AG, Munich, Germany) arranged electrically with a 90° phase difference to detect the magnetic field of the rotor magnet as quadrature analog sine and cosine signals. Angular signals were acquired at 5 kHz. Displacement and velocity were calculated at 500 Hz, and smoothed repetition-level information was transmitted at 10 Hz via the MOTY V application (version 2.0.6) using Bluetooth Low Energy. The analog sensor outputs and data interface provided an overall resolution of 12 bits, while the system-level displacement and velocity output resolutions were 1 mm and 1 mm/s, respectively. Because displacement was derived through incremental integration, the measurement principle imposed no cumulative displacement limit; the effective operating range was determined by cable length and the allowable movement velocity of the equipment.

The cable was wound around a pulley directly coupled to the motor shaft, with no gearbox or reduction gear. Therefore, the sensor-detected motor rotation and pulley rotation corresponded in a 1:1 ratio, and no gear backlash was introduced. The effective pulley radius was 0.04 m. Cable displacement was calculated as follows:

*x* = *rθ* (1)

where *x* is cable displacement, *r* is the effective pulley radius, and *θ* is the unwrapped mechanical rotation angle in radians.

Cable linear velocity was estimated from the observer-derived mechanical angular velocity and the effective pulley radius, rather than measured directly from bar displacement over time:

*v* = *rω* (2)

where *v* is cable linear velocity and *ω* is the observer-estimated mechanical angular velocity. This estimate represents cable velocity along the direction of cable movement and does not constitute a direct three-dimensional measurement of bar motion.

Raw-signal processing included acquisition and offset correction of the sine and cosine signals, arctangent-based angle calculation, phase unwrapping, and conversion to cable displacement. Angular velocity was estimated using a position-tracking observer rather than direct numerical differentiation. A first-order low-pass filter was applied at the angular-velocity stage, followed by fixed-window moving-average smoothing before transmission of the velocity output at 10 Hz via Bluetooth Low Energy (BLE). The observer and filtering parameters were selected to preserve the frequency range associated with the exercise movements. Exact cutoff frequencies and observer gains are proprietary manufacturer parameters and are not publicly disclosed. At the start of the machine’s use, the cable returns automatically to a mechanical reference position that is defined as zero displacement. During data collection, the initial angle for sensor phase alignment was stored in the firmware.

Repetition boundaries were identified from changes in cable-displacement direction together with position- and velocity-based criteria intended to suppress false detections caused by small oscillations. Cable payout was classified as the concentric phase and cable return as the eccentric phase. Mean concentric velocity was calculated by the application as the arithmetic mean of the absolute velocity samples transmitted at 10 Hz during the identified concentric phase. The recorded values were converted from cm/s to m/s for analysis. Exact numerical thresholds for repetition detection are proprietary manufacturer parameters and were not available for public disclosure.

Before the present study, MOTY V underwent independent system-level performance testing by the Korea Construction Equipment Technology Institute (KOCETI; report KCT25-GES-E034), including resistance implementation, commanded-speed maintenance, torque-response time, and operating-life testing. These tests were employed only to support equipment characterization and did not evaluate human repetition-velocity measurement or agreement with GymAware RS.

### 2.4. Comparison and Simultaneous Measurement Configuration

GymAware RS, a commercial tether-based linear position transducer, was used as the comparison device. The MOTY V exercise bar was not constrained by vertical guide rails, unlike a Smith-machine bar, and allowed anterior–posterior and mediolateral movement in addition to vertical displacement. The GymAware RS tether was attached to the MOTY V exercise bar so that both systems measured the same repetitions simultaneously. Thus, measurements from MOTY V and GymAware RS were not collected in separate sets or at different times. GymAware RS sampled at 50 Hz, and its manufacturer-defined repetition-level metric, “mean velocity,” was used. For consistency, MOTY V mean concentric velocity and GymAware RS mean velocity are referred to as repetition velocity or velocity. Identical internal repetition segmentation, filtering, and velocity-calculation algorithms were not assumed for the two systems. The MOTY V firmware version was 4.0.6. GymAware RS was used with the GymAware V2 application (version 5.4.5) for iOS/iPadOS.

### 2.5. Estimated 1RM Assessment

Deadlift and squat 1RM values used to prescribe the experimental loads were estimated rather than measured directly. On a previous day, a submaximal repetitions-to-failure assessment was conducted using the load-setting function of MOTY V. The warm-up consisted of upper- and lower-limb and trunk stretching followed by one minute of light running. The experiment began with approximately 70–80% of the load normally used by the participant in training. Participants continued until they could no longer complete an additional repetition with correct form. The estimated 1RM was calculated using the Brzycki equation [13]:

(3) $$\mathrm{Estimated}\;1\mathrm{RM}=\frac{\mathrm{Load}}{1.0278-0.0278\times \mathrm{Repetitions}}$$

where *load* is the resistance used and *Repetitions* is the number of completed repetitions at that load. Deadlift and squat estimated 1RM assessments were conducted on the same day.

### 2.6. Experimental Procedures

On measurement day, participants performed deadlifts followed by squats. Loads for each exercise were administered in a fixed ascending order of 40%, 60%, and 80% of the estimated 1RM. These three submaximal conditions provided lower, intermediate, and higher relative-load levels for examining inter-device agreement across different prescribed exercise intensities. They were not intended to characterize the full load–velocity relationship or the complete operating range of either device. Ten repetitions were planned for each exercise-load condition, yielding 900 planned paired repetitions (15 participants × 2 exercises × 3 loads × 10 repetitions). Participants were asked to perform the concentric phase of each repetition with maximal velocity. An acceptable deadlift repetition was in an upright position with a complete hip extension; an acceptable squat repetition was after hip and knee extension was completed on returning to the standing position.

Recovery was not standardized to a fixed duration. After completing a set, each participant moved to the end of the participant order and recovered while the other participants performed their sets. The same rotation-based recovery procedure was used between exercises. Exercise order and load order were not randomized.

One participant reported low-back pain after completing seven repetitions in the squat at 80% of estimated 1RM and discontinued that condition. The precise onset of discomfort during the set was not determined. The remaining three repetitions were not performed, and no measurements were generated for them. The seven completed paired repetitions were retained in the primary analysis, resulting in 897 paired observations. No missing values were imputed, and all 15 participants remained in the primary analysis.

### 2.7. Data Processing and Outcomes

The velocities of MOTY V and GymAware RS were paired using repetition identifiers. The between-device difference was defined as MOTY V–GymAware RS; therefore, a positive value would indicate a higher MOTY V velocity. The primary outcomes were the repeated-measures model-based mean bias and 95% limits of agreement. Secondary outcomes were the mean absolute error, root mean square error, Pearson correlation, set-level absolute-agreement intraclass correlation coefficient (ICC), and proportional bias. Set-level analysis used participant-by-exercise-by-load means, yielding 90 paired set-level observations.

### 2.8. Statistical Analysis

Statistical analyses were performed using Python 3.13.5 and statsmodels 0.14.6. Continuous variables are presented as mean ± standard deviation. Each participant contributed repeated velocity measurements under six exercise–load conditions. Because observations from the same participant were correlated, participant-specific random intercepts were included in the mixed-effects models of between-device differences to account for within-participant dependence. Repeated-measures Bland–Altman analysis was performed using variance components for participants, participant-by-exercise-by-load sets, and repetition-level residual error. The mean bias and its 95% confidence interval were estimated from the mixed-effects model, and the 95% limits of agreement were calculated as the estimated mean bias ± 1.96 times the square root of the sum of these variance components [14,15].

To test whether mean bias varied by exercise or load, the between-device difference was modeled as the dependent variable, with exercise, load, and the exercise-by-load interaction as fixed effects. The proportional bias was evaluated in a separate mixed-effects model by regressing the between-device difference on the mean of the two device measurements. A log-scale sensitivity analysis examined error proportional to measurement magnitude. The analysis included the repetition-level mean absolute error, root mean square error, and Pearson correlation. Absolute agreement was evaluated at the set level rather than by treating each repetition as an independent observation. Repetition-level measurements were averaged within each participant-by-exercise-by-load condition, resulting in 90 paired set means. A two-way random-effects, absolute-agreement, single-measure ICC [ICC(A,1)] was calculated for these set-level means [16]. Because each participant contributed six correlated exercise–load conditions, the 95% CI for the overall ICC was obtained using 5000 participant-level cluster bootstrap resamples, preserving the repeated-measures structure within participants. As an exploratory descriptive analysis, repeated-measures limits of agreement were also estimated separately for each relative-load level, using the same variance-components approach to account for participant-level and exercise-set-level clustering. Two post hoc sensitivity analyses were conducted to assess the influence of potentially discomfort-affected observations. The first excluded the seven completed repetitions from the affected squat condition at 80% of estimated 1RM (*n* = 890), and the second excluded all observations from the participant who reported low-back pain (*n* = 840). The same agreement-analysis procedures were applied to both datasets, and their results were compared with those of the primary analysis. Statistical significance was set at *p* < 0.05.

## 3. Results

### 3.1. Analysis and Descriptive Statistics

As noted, all 15 participants were included in the final analysis. Of 900 planned paired repetitions, three unperformed repetitions in the squat at 80% of estimated 1RM were absent, leaving 897 paired repetitions. Every performed repetition with generated data was successfully paired between MOTY V and GymAware RS. The deadlift conditions at 40%, 60%, and 80% and the squat conditions at 40% and 60% each contained 150 paired repetitions; the squat condition at 80% contained 147. Repetition-level mean velocity was 0.7102 ± 0.1496 m/s for MOTY V and 0.6816 ± 0.1410 m/s for GymAware RS. The unadjusted arithmetic mean difference was +0.0286 m/s. Condition-specific repetition velocities and mean between-device differences are presented in Table 2.

### 3.2. Inter-Device Agreement and Measurement Error

**Figure 1:**
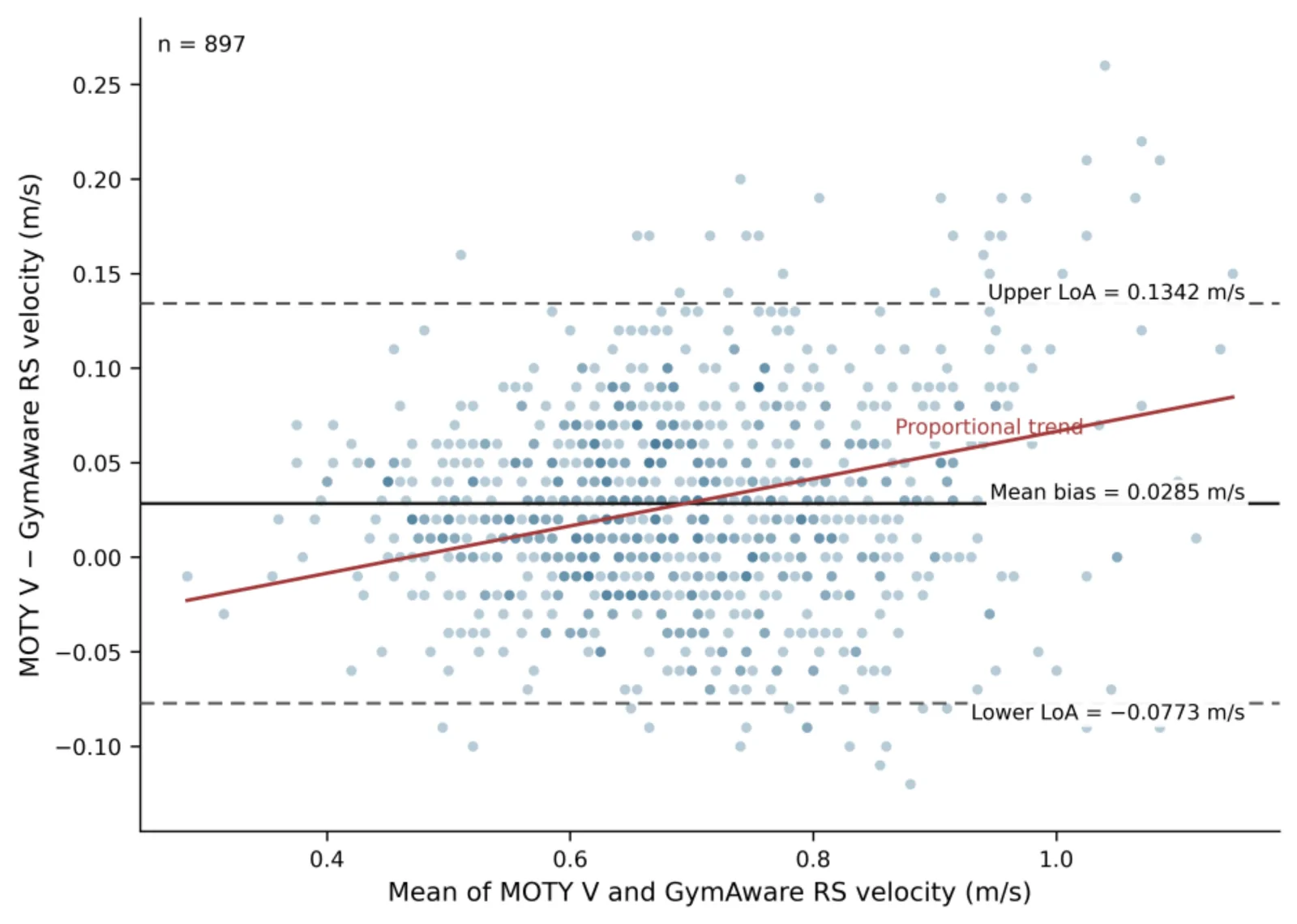
Bland–Altman analysis of repetition-velocity measurements obtained simultaneously from MOTY V and GymAware RS. Each point represents one paired repetition (*n* = 897). The solid horizontal line indicates the mean bias (MOTY V–GymAware RS; 0.0285 m/s), and the dashed horizontal lines indicate the repeated-measures 95% limits of agreement (−0.0773 to 0.1342 m/s). The fitted line illustrates the proportional trend in measurement differences across the observed velocity range.

**Figure 2:**
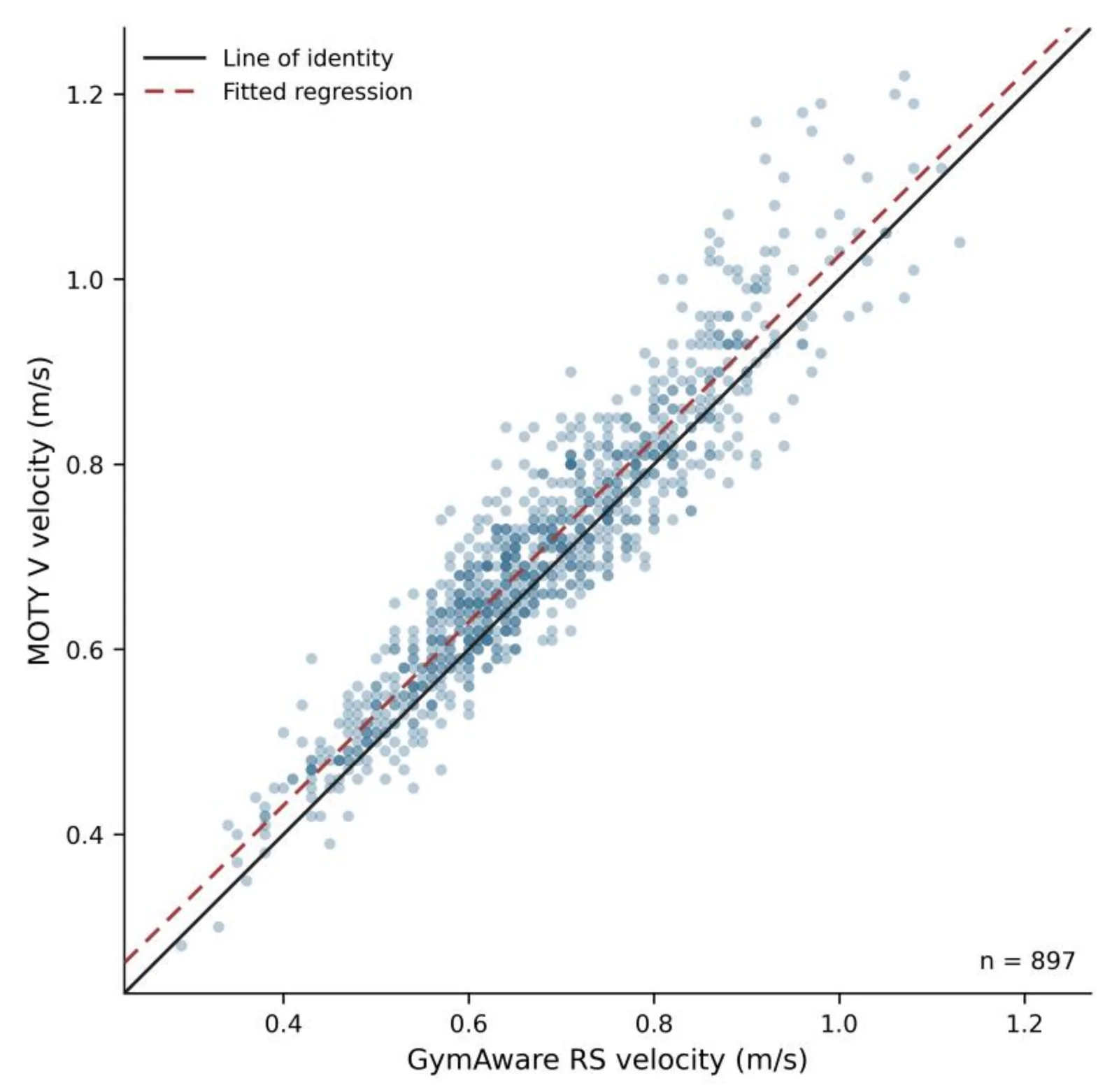
Scatter plot showing the association between repetition-velocity measurements obtained simultaneously from MOTY V and GymAware RS across all paired repetitions (*n* = 897). The solid diagonal line represents the line of identity (y = x), and the dashed fitted line summarizes the observed linear association. Agreement should be interpreted using the Bland–Altman analysis shown in Figure 1.

The mixed-effects mean bias was +0.0285 m/s (95% CI, 0.0216–0.0353), and the repeated-measures 95% limits of agreement ranged from −0.0773 to +0.1342 m/s. Repetition-level mean absolute error was 0.0467 m/s, root mean square error was 0.0609 m/s, and the Pearson correlation was *r* = 0.933. The repeated-measures Bland–Altman plot is shown in Figure 1, and the repetition-level association is displayed in Figure 2. Across the 90 participant-by-exercise-by-load set means, velocity was 0.7093 ± 0.1363 m/s for MOTY V and 0.6809 ± 0.1303 m/s for GymAware RS; the mean difference was +0.0284 m/s. The set-level two-way random-effects, absolute-agreement, single-measure ICC(A,1) was 0.958 (95% CI, 0.929–0.973), with the CI obtained by participant-level cluster bootstrap. The sensitivity analyses yielded results similar to those of the primary analysis. Excluding the seven repetitions from the affected squat condition resulted in a mean bias of +0.0290 m/s, 95% limits of agreement of −0.0764 to +0.1343 m/s, and an ICC of 0.957. Excluding all observations from the affected participant yielded a mean bias of +0.0290 m/s, limits of agreement of −0.0761 to +0.1341 m/s, and an ICC of 0.955. Significant positive proportional bias persisted in both analyses (β = 0.118 and 0.122, respectively; both *p* < 0.001). These results indicate that the overall agreement estimates were not materially influenced by the inclusion of the affected observations (Supplementary Table S1). In the exploratory load-stratified analysis, the repeated-measures 95% limits of agreement were −0.0821 to +0.1438 m/s at 40% of estimated 1RM, −0.0815 to +0.1344 m/s at 60%, and −0.0679 to +0.1244 m/s at 80%. The corresponding widths were 0.2259, 0.2159, and 0.1923 m/s, respectively, with the widest descriptive interval observed at 40% of estimated 1RM. Overall inter-device agreement and error statistics are summarized in Table 3.

### 3.3. Effects of Exercise and Load on Mean Bias

In the mixed-effects model of between-device differences, the exercise-by-load interaction (*p* = 0.82) and the main effects of exercise (*p* = 0.85) and load (*p* = 0.80) were all non-significant. Condition-specific mean differences ranged narrowly from +0.024 to +0.033 m/s.

### 3.4. Proportional Bias and Log-Scale Sensitivity Analysis

The mixed-effects model using the mean of the two device measurements as the independent variable showed significant positive proportional bias (*β* = 0.125, 95% CI, 0.082–0.167; *p* < 0.001). Thus, a 0.10 m/s increase in the mean velocity of the two devices was associated with an approximately 0.012 m/s increase in the MOTY V–GymAware RS difference. In the log-scale sensitivity analysis, the geometric mean ratio of MOTY V to GymAware RS was 1.041 (95% CI, 1.031–1.051) and the ratio-scale 95% limits of agreement were 0.900–1.205.

## 4. Discussion

### 4.1. Principal Findings

This study compared repetition-velocity measurements obtained from a motor-integrated magnetic sensing system with those obtained from GymAware RS. Both devices were attached to the same exercise bar and simultaneously measured the same repetitions. The principal findings were as follows. First, the devices showed a strong repetition-level correlation (*r* = 0.933) and high set-level absolute agreement, with an ICC of 0.958 (95% CI, 0.929–0.973). Second, repeated-measures Bland–Altman analysis showed a mean bias of +0.0285 m/s (MOTY V-GymAware RS), with 95% limits of agreement of −0.0773 to +0.1342 m/s. Third, exercise, relative load, and their interaction had no significant effect on mean bias. Fourth, the between-device difference increased with the mean velocity of the two devices, indicating significant proportional bias. Therefore, the high association and absolute agreement should be interpreted together with small positive systematic bias and proportional bias, rather than as evidence of direct interchangeability.

### 4.2. Measurement Agreement and Systematic Bias

The repetition-level Pearson correlation indicated that MOTY V velocity closely tracked the overall variation in GymAware RS velocity. The set-level absolute-agreement ICC of 0.958 (95% CI, 0.929–0.973) similarly indicated a high absolute agreement between the means of the paired set. The repeated-measures model-based mean bias was +0.0285 m/s (95% CI, 0.0216–0.0353), indicating that MOTY V yielded repetition velocities approximately 0.03 m/s higher than that of GymAware RS on average across the observed exercises and loads. Repetition-level mean absolute error and root mean square error were 0.0467 and 0.0609 m/s, respectively. However, the mean bias alone did not describe the range of differences for the individual repetitions. The repeated-measures 95% limits of agreement extended from −0.0773 to +0.1342 m/s, showing that paired differences were more widely distributed than the average bias. Therefore, the mean bias of approximately +0.029 m/s should not be interpreted as constant for every repetition.

In velocity-based resistance training, movement-velocity thresholds may be used to regulate exercise loads or determine set termination based on velocity loss [1,2]. Although the average between-device difference was approximately +0.03 m/s, the repeated-measures 95% limits of agreement ranged from −0.0773 to +0.1342 m/s, indicating substantially greater variation in individual paired repetitions. Consequently, repetitions near a predefined velocity threshold could be classified differently depending on the measurement device, potentially affecting load adjustment or set-termination decisions. The significant proportional bias further indicates that a fixed correction of approximately 0.03 m/s may not be sufficient across the observed velocity range. Because no application-specific acceptable-error threshold was established a priori, the practical acceptability of these differences cannot be assumed for all velocity-based training applications. Previous studies examined the validity and test–retest reliability of the GymAware PowerTool in resistance exercises [5,6], whereas GymAware RS was compared separately with three-dimensional motion capture for peak velocity during snatch and clean movements [7]. In another laboratory comparison, Thompson et al. [17] reported limits of agreement of approximately −0.03 to +0.03 m/s for a GymAware device relative to three-dimensional motion capture. This provides useful context, although differences in device versions, exercises, velocity metrics, and analytical procedures preclude a direct quantitative comparison with the present limits of agreement. Moreover, the present study cannot determine how much of the observed inter-device disagreement arises from either system because no independent criterion measurement was obtained. A comparison between GymAware and another velocity device also found that strong correlations could coexist with systematic differences and limits of agreement that precluded direct interchangeability [8]. The present findings similarly demonstrate why correlation or ICC should not be used alone. A recent systematic review emphasized that validity claims for velocity-monitoring devices depend on the use of an appropriate criterion system and complementary statistical measures of measurement error and agreement [18]. In a subsequent study, Dello Stritto et al. [19] compared mean and peak velocity outputs from two linear position transducers across different movement-velocity ranges, demonstrating the importance of considering both the velocity metric and the measurement conditions when interpreting device agreement. These findings reinforce the need to interpret the present results as inter-device agreement within the tested exercises and velocity range rather than as evidence of criterion validity or universal interchangeability.

### 4.3. Proportional Bias

The positive proportional-bias coefficient indicated that the MOTY V–GymAware RS difference increased as the average measured velocity increased. A 0.10 m/s increase in the pair’s mean corresponded to an approximately 0.012 m/s increase in the between-device difference. Thus, the observed discrepancy cannot be explained solely by a fixed correction applied uniformly across the velocity range. The exploratory load-stratified analysis also showed the widest limits of agreement at 40% of estimated 1RM, although the differences between load-specific intervals were not formally tested. This descriptive pattern should be distinguished from proportional bias, which describes a change in expected between-device difference with measured velocity rather than necessarily indicating greater dispersion of errors at higher velocities.

The log-scale sensitivity analysis supported this interpretation. The geometric mean ratio of 1.041 indicated that the MOTY V values were, on average, approximately 4.1% higher than those of GymAware RS, whereas the ratio-scale 95% limits of agreement of 0.900 to 1.205 indicated variability related to measurement magnitude. These results are directionally consistent with the positive mean bias and proportional bias observed on the raw scale.

MOTY V calculates mean concentric velocity from samples assigned to the concentric phase, whereas GymAware RS provides a manufacturer-defined mean-velocity metric. Differences in sensing principles and signal processing, including sampling frequency, repetition segmentation, filtering, and velocity averaging, may have contributed to the observed systematic and proportional biases [4]. However, the study was not designed to isolate the contributions of individual hardware or processing components. Consequently, the observed bias cannot be attributed to a specific source. In future studies, if values from the two systems need to be related, a device-specific calibration or threshold-translation procedure that accounts for velocity magnitude may be more appropriate than subtracting 0.03 m/s from every MOTY V value. No calibration equation was developed or independently validated; therefore, no correction formula was established.

### 4.4. Effects of Exercise and Load

Neither the exercise-by-load interaction (*p* = 0.82) nor the main effects of exercise (*p* = 0.85) or load (*p* = 0.80) reached statistical significance. Condition-specific mean differences ranged from +0.024 to +0.033 m/s. Therefore, the average positive bias was not confined to either the deadlift or squat, or to a single relative-load condition within the tested range.

Movement velocity, displacement, and technique can vary by exercise and relative load. The absence of a significant condition effect is relevant to the potential use of equipment-integrated sensing across the tested conditions, but it does not establish identical bias or equivalence at all exercises and loads. With 15 participants and fixed exercise and load orders, the power to detect small condition-specific differences and the generalizability of the results remain limited.

Condition-specific descriptive values showed that mean deadlift velocity was higher at 60% than at 40% of estimated 1RM for both devices and remained slightly higher at 80% than at 40%. Thus, the observed velocities did not follow a monotonic load–velocity pattern. The similar pattern observed with both devices suggests that it was not unique to MOTY V measurements; however, its underlying cause cannot be determined from the present data. Potential contributing factors include error in estimated 1RM, familiarization and technique changes associated with the fixed ascending load order, differences in the initial pull and cable tension, and variation in recovery between sets. Fatigue and order effects may also have influenced condition-specific velocity measurements, although they cannot be identified as the cause of the non-monotonic pattern. These explanations remain hypothetical because the contributing factors were not measured independently. Future studies should use randomized or counterbalanced load sequences, standardized recovery periods, and additional kinematic assessments.

### 4.5. Implications of Equipment-Integrated Sensing

MOTY V derives cable displacement and velocity from magnetic measurement of motor-shaft rotation and the direct coupling between the motor and pulley. This equipment-integrated-sensor approach can provide repetition velocity without the need for an external velocity sensor attached to the exercise bar. Kinematic information generated within motor-driven or digitally controlled resistance equipment can be recorded during exercise and can support real-time feedback or velocity-based load control [11,12].

Recent developments in wearable sensors and intelligent human–machine interfaces have expanded approaches to monitoring human movement and integrating sensor information with assistive systems. For example, advances involving wearable sensors, brain–machine interfaces, and exoskeleton robotics have been reviewed by Wang et al. [20]. In contrast to wearable sensing approaches, MOTY V derives repetition-velocity information from sensors integrated directly into resistance-training equipment. These approaches represent different sensing architectures, and the present study was limited to evaluating inter-device measurement agreement rather than broader human–machine interaction or training outcomes.

Repetition-level velocity feedback can also encourage individuals to maintain maximal velocity [3]. Therefore, integrating velocity calculation and transmission into exercise equipment may simplify exercise procedures and improve access to velocity information. However, this requires consistent interpretation of the measurements. Given the observed systematic and proportional bias, a MOTY V velocity threshold should not automatically be assumed to represent the same threshold on another device. As a comparison of repetition-velocity measurement equipment, this study did not test whether real-time feedback or automated load control with MOTY V improved strength, power, fatigue, or long-term training outcomes. Therefore, the findings support the feasibility of equipment-integrated velocity measurement but do not demonstrate the effectiveness of exercise intervention using this system.

### 4.6. Study Strengths

A principal strength of this study was the simultaneous measurement of the same repetition and bar movement by both systems. If devices are evaluated in different sets or at different times, performance fluctuations and fatigue can contribute to apparent between-device differences. The simultaneous configuration reduced this biological variability and enabled direct comparison at the repetition level. The dataset contained 897 paired repetitions across two exercises and three relative loads. Repetition-level measurements were clustered within participants and exercise-load conditions, and the analyses used repeated-measures Bland–Altman and mixed-effects methods rather than treating all repetitions as independent observations. Independent system-level performance testing was also available for MOTY V. Those tests were interpreted narrowly as checks of resistance implementation, speed maintenance, response time, and operating life, rather than as evidence of absolute validity during human exercise. MOTY Inc. did not assist in participant recruitment, statistical analysis, interpretation of the results, or manuscript preparation, and both measurement systems were purchased and institutionally owned by the university laboratory.

### 4.7. Study Limitations

The study has several limitations. First, no a priori sample-size calculation was performed, and the sample comprised 15 male university students with at least six months of resistance-training experience. Although repeated measurements provided information across six exercise–load conditions, they did not increase the number of independent participants. Consequently, the precision of the agreement estimates and the ability to detect small condition-specific differences may have been limited. The findings should not be generalized directly to women, other age groups, novice lifters, or clinical populations. Second, the evaluation was restricted to deadlift and squat exercises at 40%, 60%, and 80% of estimated 1RM. This restricted interval did not encompass very light or near-maximal loading conditions, and agreement at movement velocities outside the observed range could not be determined. The measurement characteristics of other exercises and untested loads therefore remain uncertain. Third, 1RM was estimated using a submaximal repetitions-to-failure assessment rather than measured directly. Therefore, individual relative loads may have included some estimation error. Fourth, the exercise order was fixed as a deadlift followed by a squat, and loads were administered in a fixed ascending order. Recovery was rotation-based rather than standardized to a fixed duration. Fatigue, order effects, and differences in actual recovery time may have affected condition-specific velocities. Because the devices measured each repetition simultaneously, their influence on the within-repetition device comparison was partly constrained. Fifth, MOTY V provided mean concentric velocity as its output, whereas GymAware RS provided the metric it called “mean velocity.” Thus, each machine’s internal velocity calculations and repetition-segmentation algorithms may not be identical. Therefore, the observed differences may include both hardware-related differences and differences in signal processing or metric definition. Sixth, no traceable laboratory-grade three-dimensional motion-analysis system or separate criterion displacement system was used. Thus, the findings characterize concurrent measurement and agreement only between MOTY V and GymAware RS, but do not determine which system is closer to true movement velocity or whether MOTY V is more accurate than GymAware RS. Finally, one participant stopped the squat condition at 80% of estimated 1RM after seven repetitions because of low-back pain. The final three repetitions were not generated; the seven completed repetitions were retained, and missing values were not imputed, resulting in a slight imbalance in repetition counts among conditions. Because the precise onset of discomfort was not documented, the possibility that pain had already affected movement performance during the preceding repetitions cannot be excluded. This may have introduced bias in the affected condition. Nevertheless, sensitivity analyses excluding either the affected repetitions or the entire participant produced similar overall agreement estimates. These findings support the robustness of the principal results but do not establish that the retained repetitions were unaffected by discomfort.

### 4.8. Overall Interpretation

The equipment-integrated magnetic sensing system of MOTY V showed a strong association and high set-level absolute agreement with that of GymAware RS for repetition-velocity measurements during deadlift and squat exercises. The absence of a significant exercise or load effect on mean bias suggests the need for further testing of equipment-integrated velocity measurements across these conditions. Nevertheless, MOTY V velocity measured approximately 0.03 m/s higher than that of GymAware RS on average, and the between-device difference increased with velocity. Therefore, the values should not be treated as a common scale, and thresholds established with one device should not be transferred directly to the other. This warrants further validation with a laboratory-grade criterion system and evaluation of device-specific calibration or threshold translation in future independent samples.

## 5. Conclusions

Repetition velocity derived from the motor-integrated magnetic sensing system of MOTY V showed a strong association and high absolute agreement with the simultaneous GymAware RS measurements during deadlift and squat exercises. MOTY V values were approximately 0.03 m/s higher on average, although mean bias did not differ significantly by exercise or relative load. The small positive systematic bias and significant proportional bias indicate that MOTY V and GymAware RS values should not be considered identical or fully interchangeable. MOTY V has potential as an equipment-integrated system for providing repetition-velocity information, but device-specific interpretation is required.

## Figures and Tables

**Table 1:** Participant characteristics (*n* = 15).

Variable	Mean ± Standard Deviation
Age (years)	20.53 ± 1.81
Height (cm)	171.60 ± 4.34
Body mass (kg)	71.13 ± 6.97
Skeletal muscle mass (kg)	33.86 ± 2.81
Body mass index (kg/m^2^)	24.16 ± 2.23
Fat-free mass (kg)	60.06 ± 4.75
Body fat percentage (%)	16.79 ± 4.94
Estimated deadlift 1RM (kg)	111.71 ± 13.53
Estimated squat 1RM (kg)	109.63 ± 17.86

Note: Body-composition variables were measured and reported to describe the sample and were not used as predictors or outcomes in the inferential analyses of inter-device agreement. SD, standard deviation; 1RM, one-repetition maximum.

**Table 2:** Repetition velocity and mean between-device difference by exercise and relative load.

Exercise	Load	*n*	MOTY V(m/s)	GymAware RS (m/s)	Mean Difference (m/s)
Deadlift	40%	150	0.732 ± 0.150	0.699 ± 0.141	+0.033
Deadlift	60%	150	0.767 ± 0.144	0.743 ± 0.132	+0.024
Deadlift	80%	150	0.746 ± 0.112	0.718 ± 0.117	+0.027
Squat	40%	150	0.729 ± 0.141	0.700 ± 0.123	+0.029
Squat	60%	150	0.686 ± 0.098	0.658 ± 0.104	+0.029
Squat	80%	147	0.596 ± 0.112	0.567 ± 0.101	+0.029

Note: Values are shown as mean ± standard deviation. Mean differences were calculated from unrounded repetition-level data as MOTY V–GymAware RS; consequently, some values differ from simple subtraction of the displayed rounded means. Load is expressed as a percentage of estimated 1RM, one-repetition maximum.

**Table 3:** Overall inter-device agreement and error statistics for MOTY V and GymAware RS.

Analysis Level	Statistic	Result
Repetition level (*n* = 897)	Mean bias (MOTY V–GymAware RS)	+0.0285 m/s
Repetition level (*n* = 897)	95% CI for mean bias	0.0216–0.0353 m/s
Repetition level (*n* = 897)	Repeated-measures 95% limits of agreement	−0.0773 to +0.1342 m/s
Repetition level (*n* = 897)	Mean absolute error	0.0467 m/s
Repetition level (*n* = 897)	Root mean square error	0.0609 m/s
Repetition level (*n* = 897)	Pearson correlation	*r* = 0.933
Set level (*n* = 90)	Absolute-agreement ICC(A,1)	0.958 (95% CI, 0.929–0.973)

Note: CI, confidence interval; ICC, intraclass correlation coefficient.

## Data Availability

The data presented in this study are available from the corresponding author upon request. The data are not publicly available owing to privacy considerations related to identifiable participant-level exercise performance records.

## Supplementary Materials

The following supporting information can be downloaded at https://www.mdpi.com/article/10.3390/s26206466/s1, Table S1: Post hoc sensitivity analyses excluding potentially discomfort-affected observations.
