# Supplementary material for: Comparing Repetition-Velocity Output from a Motor-Integrated Magnetic Sensing System with That of a Linear Position Transducer Across Resistance Exercises and Loads: A Human Trial Experiment
Source: Sensors (Basel). 2026 Oct 13;26(20):6466. doi: 10.3390/s26206466 (PMC13709254; doi:10.3390/s26206466)
Supplement: Supplementary file 1 [file sensors-26-06466-s001.zip › sensors-4599407-supplementary.pdf]

Supplementary Materials

Table S1. Post hoc sensitivity analyses excluding potentially discomfort-affected observations.

Statistic	Primary analysis (all observations)	Exclude affected 7 repetitions	Exclude affected participant
Participants (n)	15	15	14
Paired repetitions (n)	897	890	840
Paired sets (n)	90	89	84
Mean bias (m/s)	+0.0285	+0.0290	+0.0290
95% LoA, lower (m/s)	-0.0773	-0.0764	-0.0761
95% LoA, upper (m/s)	+0.1342	+0.1343	+0.1341
ICC(A,1)	0.958	0.957	0.955
MAE (m/s)	0.0467	0.0467	0.0466
RMSE (m/s)	0.0609	0.0610	0.0609
Proportional bias (β)	0.125	0.118	0.122
Proportional bias (p)	<0.001	<0.001	<0.001

Note. The primary analysis included 897 simultaneous repetition pairs from 15 participants. The first sensitivity analysis excluded seven completed squat repetitions at 80% of estimated 1RM from the participant who subsequently reported low-back pain. The second excluded all 57 observations from that participant. No missing values were imputed.

Mean bias is MOTY V minus GymAware RS. The 95% limits of agreement (LoA) were obtained from a repeated-measures Bland–Altman model accounting for participant-, set-, and repetition-level variability. ICC(A,1) was calculated from participant-by-exercise-by-load set means; proportional-bias slopes were estimated using mixed-effects models.

Abbreviations: ICC, intraclass correlation coefficient; LoA, limits of agreement; MAE, mean absolute error; RMSE, root mean square error; 1RM, one-repetition maximum.
